# Further available immunization option to prevent pneumococcal disease

**DOI:** 10.12688/f1000research.5990.1

**Published:** 2015-01-07

**Authors:** Ivo Vojtek, Bernard Hoet

**Affiliations:** 1 GSK Vaccines, Wavre, 1300, Belgium

**Keywords:** PHiD-CV, efficacy, post-marketing surveillance

## Abstract

In their recent review, Charles Feldman and Ronald Anderson provide an overview of various clinical aspects of pneumococcal infections. We would like to complete this report by providing some additional information on a widely-used immunization option, which was not originally mentioned in the article. The protein D pneumococcal conjugate vaccine (PHiD-CV) has been pre-approved by WHO and its impact is supported by real-life data from the regions of its use.

## Correspondence

We write in response to the report by Charles Feldman and Ronald Anderson about the recent advances in the understanding of *Streptococcus pneumoniae* infections ^1^.

While this article provided an informative and complete review of the current burden of the disease, pathogenesis and therapeutic options, we have noted a significant omission in the chapter dealing with available immunization strategies, which did not mention the WHO prequalified Pneumococcal Nontypeable *Haemophilus influenzae* Protein D Conjugate Vaccine (PHiD-CV; GSK Vaccines, Belgium). This vaccine is currently licensed in more than 125 countries with more than 200 million doses distributed as of August 2014 and is used in vaccination programmes in more than 40 countries or regions.

 We feel it is important that health care professionals are made aware of the available evidence supporting the use of this vaccine in order that they are able to make an informed choice about the best care for their patients, and therefore we provide additional information to supplement the review article. It is the only modern pneumococcal conjugate vaccine with impact on invasive pneumococcal disease, pneumonia and acute otitis media that has been proven in two pivotal randomized controlled efficacy trials performed in Finland and Latin America ^2– 4^. Thanks to its world-wide use, there is also a plethora of post-marketing and epidemiology data spanning five continents, recently reviewed by Plosker ^8^, that proves its impact on the pneumococcal disease and makes it a worth-while alternative to the pneumococcal conjugate vaccine PCV13 which the health care community should be made aware of ^5– 7^. We have summarized the main effectiveness and impact data in Table 1.

**Table 1.** Summary of the main effectiveness and impact data of PHiD-CV. IPD: Invasive Pneumococcal disease; VE: vaccine efficacy; RR: relative rate reduction.

Randomized Clinical Trials			
Region	Indication		
	Invasive Pneumococal Disease	Acute Otitis Media	Consolidated pneumonia
Finland	Vaccine serotype - 3+1 **VE=100%** (95%CI : 83, 100) ^2^ Vaccine serotype - 2+1 **VE=92%** (95% CI: 58, 100) ^2^	X	X
	Any Serotype - 3+1/2+1 **VE=93%** (95% CI: 75, 99) ^2^	X	**VE=44%** (95% CI: 24, 59) ^4^
Latin America	Vaccine serotype - 3+1 **VE=100%** (95% CI: 77, 100) ^3^ Any Serotype - 3+1 **VE=67%** (95% CI: 22, 86) ^3^	Vaccine serotype **VE=70%** (95% CI: 30, 87) ^3^ Clinical Diagnosed **VE=19%** (95% CI: 4, 31) ^3^	**VE=26%** (95% CI: 8, 40) ^3^
**Impact and surveillance data on Invasive Pneumococcal Disease**			
Quebec (case- controlled study)	Vaccine-type (+6A) VE=99% (95% CI: 79, 100) ^5^ 19A IPD **VE=67%** (95% CI: 8, 88) ^5^ All IPD **VE=75%** (95% CI: 53, 79) ^5^		
Brazil (case- controlled study)	Vaccine type **VE=84%** (95% CI: 66, 92) ^6^ 19A **VE=82%** (95% CI: 11, 96) ^6^		
Finland (time series analysis)	Vaccine type **RR=92%** (95% CI: 85, 96) ^7^ 19A **RR=77%** (95% CI: 41, 93) ^7^ All IPD **RR=80%** (95% CI: 72, 86) ^7^		
