# Supplementary material for: Further available immunization option to prevent pneumococcal disease
Source: F1000Res. 2015 Jan 7;4:3. [Version 1] doi: 10.12688/f1000research.5990.1 (PMC4376167; doi:10.12688/f1000research.5990.1)
Supplement: Peer review history

# Peer review: Referee response for version 1

**DOI:** 10.5256/f1000research.6408.r7587

**Published:** 2015-03-23

**Authors:** Marco Safadi

**Affiliations:** 1 Department of Pediatrics, Santa Casa University, São Paulo, Brazil

The correspondence article *“Further available immunization option to prevent pneumococcal disease”* from Vojtek & Hoet provides relevant information regarding one of the currently available pneumococcal conjugate vaccines. This information was missing in the review article (Recent advances in our understanding of *Streptococcus pneumoniae* infection ) written by Feldman & Anderson. 

The correspondence article is well written and should be considered for indexing.

I have only minor comments:

- In the second paragraph, when mentioning that the PHiD-CV vaccine is currently licensed in more than 125 countries, the authors should make clear that the vaccine **is licensed** **for active immunization against invasive disease, pneumonia, and acute otitis media (AOM) caused by *S. pneumoniae* in infants and young children up to 5 years of age**.
- In the third paragraph, when mentioning that the PHiD-CV vaccine is a worth-while alternative to the pneumococcal conjugate vaccine PCV13, the authors should add: … **in children younger than 5 years of age**.
- In Table 1, when showing the different study results observed with the PHiD-CV vaccine, **the authors should specify which results are vaccine efficacy data and which are vaccine effectiveness data**. 

I have read this submission. I believe that I have an appropriate level of expertise to confirm that it is of an acceptable scientific standard.

# Peer review: Referee response for version 1

**DOI:** 10.5256/f1000research.6408.r7220

**Published:** 2015-01-20

**Authors:** Paola Marchisio

**Affiliations:** 1 Department of Pathophysiology and Transplantation, University of Milan, Milan, Italy

I read with great interest the letter of Vojtek and Hoet. Well done and necessary. I have some remarks and suggestions:

- In the abstract the term “pre-approved” is not clear to all the readers. The exact date of approval would be useful because it could inform the readers that PHiD-CV has a long and great history.
- Page 2: “prequalified” is not clear (see above)
- Page 2 , second column, third line. I would add “evidence based” before “informed choice” in order to stress the big amount of available rigorous data.
- Page 2, 11th line: I would say “proves its **beneficial** impact…”
- It should be underlined that PHiD-CV is approved for use in children younger  than  5 years of age (that could be one the reason why it was not quoted in the paper of Feldam which focuses mostly on adult/elderly patients)
- In the table I would add “schedule” before “3+1”  or “2+1” . In the current version one may read “minus 3 plus 1” and may misunderstand.  

I have read this submission. I believe that I have an appropriate level of expertise to confirm that it is of an acceptable scientific standard.
